# Cognition and Health-Related Quality of Life After aSAH: The Role of Objective and Subjective Impairment

**DOI:** 10.3390/neurolint18030062

**Published:** 2026-03-23

**Authors:** Angelka Pešterac-Kujundžić, Una Nedeljković, Ivana Sretenović, Aleksandar Milosavljević, Dragoslav Nestorović, Vojislav Bogosavljević, Ivan Vukašinović

**Affiliations:** 1 College of Health Sciences, Academy of Applied Studies Belgrade, Cara Dušana 254, 11000 Belgrade, Serbia; 2 Faculty of Medicine, University of Belgrade, 11000 Belgrade, Serbia; unaned@gmail.com (U.N.);; 3 Center for Physical Medicine and Rehabilitation, University Clinical Center of Serbia, 11000 Belgrade, Serbia; 4 Faculty of Special Education and Rehabilitation, University of Belgrade, 11000 Belgrade, Serbia; 5 Clinic for Neurosurgery, University Clinical Center Kragujevac, 34000 Kragujevac, Serbia; 6 Center for Radiology and Magnetic Resonance Imaging, University Clinical Center of Serbia, 11000 Belgrade, Serbia; 7 Clinic for Neurosurgery, University Clinical Center of Serbia, 11000 Belgrade, Serbia

**Keywords:** aneurysmal subarachnoid hemorrhage, objective cognitive performance, subjective cognitive complaints, health-related quality of life, long-term outcomes

## Abstract

**Objectives:** Survivors of mild-grade aneurysmal subarachnoid hemorrhage (aSAH) often achieve favorable neurological recovery, yet many continue to experience cognitive difficulties and reduced health-related quality of life (HRQoL). The relative contribution of objectively measured cognition and subjective cognitive complaints to long-term HRQoL in this population remains insufficiently clarified. **Methods**: This prospective cohort study assessed objective and subjective cognitive functioning one year after mild-grade aSAH (Hunt & Hess I–II) and examined their unique contributions to HRQoL. Forty endovascularly treated aSAH survivors and 80 neurologically healthy controls, matched for sex, age, and educational level, were assessed 12–14 months post-ictus using the Montreal Cognitive Assessment (MoCA), Cognitive Failures Questionnaire (CFQ), and SF-36. **Results**: Compared with controls, patients demonstrated significantly lower MoCA scores, with cognitive impairment present in 42.5% of cases, as well as reduced HRQoL. In multivariate regression analyses adjusted for demographic, clinical, and affective covariates, subjective cognitive complaints (CFQ) remained independently associated with the mental component summary score of the SF-36 (*β* = −0.47, *p* = 0.002). Objective cognitive performance (MoCA) was not associated with the SF-36 component summary scores but showed weaker, domain-specific associations in exploratory analyses. The correlation between MoCA and CFQ was weak (*ρ* = −0.33), indicating a dissociation between these two measures. **Conclusions**: One year after mild-grade aSAH, subjective cognitive complaints contribute to mental HRQoL above and beyond the influence of affective symptoms. These findings highlight a clinically relevant dissociation between perceived and objectively measured cognition and support the importance of incorporating patient-reported cognitive difficulties into long-term outcome assessment and rehabilitation planning.

## 1. Introduction

Over time, the concept of quality of life has evolved from philosophical debates on what constitutes a good life to a multidisciplinary construct encompassing physical, psychological, and social dimensions of functioning [1]. Importantly, an individual may experience poor health yet report a high subjective quality of life, and vice versa [2]. Cognitive functions therefore play a central role in quality of life, as they influence not only everyday functioning but also the way individuals perceive and evaluate their own life circumstances. Cognitive dysfunction is a common sequela across neurological disorders and can have long-lasting consequences for work participation, social relationships, and daily activities, thereby affecting multiple domains of quality of life [3]. This is particularly relevant for individuals who survive aneurysmal subarachnoid hemorrhage (aSAH), a population that differs from other stroke groups by its younger age profile and relatively lower levels of physical disability. Although 55% of individuals with aSAH regain independent functioning [4], many fail to return to their premorbid level of personal, social, and professional functioning.

Cognitive impairment is a common consequence of aneurysmal subarachnoid hemorrhage (aSAH), with deficits most frequently observed in the domains of memory, executive function, and language [5]. Contemporary studies confirm the high prevalence of these deficits, indicating that over 70% of patients exhibit impairment in at least one cognitive domain one year post-hemorrhage, even among those with full functional recovery [6]. These functions are essential for everyday activities and social participation, making their disruption highly relevant for functional outcome. Accordingly, cognitive difficulties are strongly associated with unsuccessful return to previous social and occupational roles [7,8].

These cognitive deficits directly impact long-term well-being. Approximately one-third of survivors experience markedly reduced health-related quality of life (HRQoL) one year after hemorrhage, with cognitive difficulties being one of the key contributors to poor long-term outcome [6,9,10]. Evidence further indicates that objectively measured global cognitive impairment is an important predictor of reduced HRQoL and emotional functioning, whereas the additional contribution of individual cognitive domains becomes limited once overall cognitive status is taken into account [11].

However, health-related quality of life after aSAH is not solely determined by objectively measured cognitive capacity. Research demonstrates that both subjective cognitive complaints and objectively measured cognitive functioning are significant independent predictors of long-term HRQoL, with each contributing unique variance even after accounting for demographic and clinical factors [12]. This finding underscores a critical principle: quality of life is influenced not only by what individuals can or cannot do, but also by how they perceive, interpret, and evaluate their own functioning. Consequently, the subjective experience of cognitive ability may be equally, or in some cases more, relevant to HRQoL than objectively measured impairment. Multivariate models of long-term outcome confirm this finding, indicating that subjective cognitive complaints, together with psychological factors like mood and fatigue, explain a substantial proportion of variance in overall quality of life [13]. This overlap highlights the complex interplay between cognitive perceptions, emotional distress, and self-reported health status.

Long-term studies demonstrate that objectively measured cognitive performance is not significantly related to patients’ cognitive complaints, whereas these complaints are strongly associated with long-term psychological well-being (including anxiety and depression) and, critically, with greater difficulty returning to work after aSAH [14]. This pattern underscores that subjective complaints capture a dimension of recovery distinct from neuropsychological test scores. Furthermore, subjective cognitive complaints independently predict participation restrictions and satisfaction with participation [15], as well as overall quality of life [13].

Despite the established importance of both dimensions, the relative and combined contributions of subjective cognitive complaints and objectively measured cognition to HRQoL after aSAH remain insufficiently understood. Clarifying this relationship is particularly important, as it mirrors the observed dissociation between objective and subjective cognition. It is particularly relevant in patients with favorable neurological outcomes, in whom cognitive deficits may remain masked by functional independence, potentially leading to under-recognition and undertreatment of an important determinant of poor quality of life. Differentiating subjective and objective components of cognitive functioning is therefore essential for accurately interpreting their impact on HRQoL in this population. Therefore, the present study aimed to assess both objective cognitive performance and subjective cognitive complaints in a cohort of aSAH survivors with favorable neurological outcomes, and to examine their unique and combined contributions to different dimensions of HRQoL one year after ictus.

## 2. Material and Methods

### 2.1. aSAH Patients and Procedure

Ethical approval for the study was obtained from the Institutional Ethics Committee (No. 1039/3, 26 October 2022), and written informed consent was obtained from all participants. Consecutive patients with aneurysmal subarachnoid hemorrhage who underwent endovascular embolization at the Clinic for Neurosurgery, University Clinical Center of Serbia, were screened for eligibility between November 2022 and December 2023. Eligible patients were adults (≥18 years) who presented with mild clinical grades (Hunt & Hess grades I–II) in the acute phase, were fluent in Serbian, and resided in Serbia throughout the study period. Patients with traumatic or non-aneurysmal SAH, previous cerebrovascular events, severe comorbidities likely to interfere with follow-up, or residence abroad were not considered for participation.

The study was designed as a prospective observational cohort of mild-grade aSAH patients and compared them with matched healthy controls. In accordance with routine clinical care, all eligible individuals were scheduled for a standard one-year outpatient follow-up visit. Patients were evaluated during the routine one-year follow-up visit (12–14 months post-ictus). At the visit, cognitive screening was performed by a licensed clinician, and patients completed questionnaires with assistance provided for item clarification when needed.

For each patient, two neurologically healthy controls were recruited from the patient’s social environment and local community and matched on sex, age, and educational level (low/intermediate vs. high). This matching procedure was carried out at the group level (frequency matching), meaning that controls were not individually paired with specific patients. It was performed to minimize the influence of demographic variables known to affect cognitive performance and quality of life outcomes. Inclusion criteria were fluency in Serbian and absence of neurological or psychiatric disease; exclusion criteria included prior cerebrovascular events, malignancy, NYHA class III–IV cardiovascular disease, and severe physical disability.

### 2.2. Measures and Instruments

Baseline clinical data (age at ictus, sex, level of education, Hunt and Hess grade at admission, aneurysm location, and length of hospital stay) were obtained from medical records during acute hospitalization. Hemorrhage severity at ictus was graded using the Hunt and Hess scale, a five-level clinical measure of initial neurological status ranging from mild symptoms (grade I) to coma (grade V) [16]. Clinical variables were defined as follows. Ischemia was defined as delayed cerebral ischemia documented during the acute hospitalization, based on clinical deterioration and/or new ischemic lesions on neuroimaging (CT or MRI) not attributable to other causes. Hydrocephalus was defined as radiologically confirmed ventricular enlargement requiring clinical management during the acute phase. Aneurysm location was categorized as anterior versus posterior circulation according to standard vascular classification based on angiographic findings. Posterior circulation included aneurysms of the vertebrobasilar system, whereas anterior circulation included aneurysms arising from the internal carotid, anterior cerebral, and middle cerebral artery systems. Sociodemographic variables (marital status, living situation, and employment status) were collected during the one-year follow-up visit. Functional disability at one year was evaluated using the modified Rankin Scale (mRS), which rates global disability from 0 (no symptoms) to 6 (death), with lower scores indicating greater functional independence [17]. At the one-year visit, participants completed cognitive and quality-of-life assessments administered face-to-face by the investigator.

Cognitive performance was evaluated using the Serbian version of the Montreal Cognitive Assessment (MoCA; MoCA Cognition Inc., Greenfield Park, QC, Canada) [18]. The MoCA is a 30-point screening instrument covering attention, memory, executive functions, language, visuospatial abilities, and orientation. In accordance with standard MoCA instructions, one point was added to the total score for participants with ≤12 years of education to correct for education effects. Following recommendations for cognitive screening after aSAH [19], a score of ≤22 was used to define cognitive impairment for the purpose of this study. Evidence from systematic reviews indicates that lower thresholds than the original cutoff of 26 improve diagnostic accuracy in aSAH populations by reducing false-positive classifications. Consequently, for subgroup analyses, participants were dichotomized as cognitively impaired (MoCA ≤ 22) or cognitively preserved (MoCA > 22). All assessments were administered by a trained investigator licensed to use the MoCA, following standardized instructions.

Subjective cognitive complaints were assessed using the self-report Cognitive Failures Questionnaire (CFQ) [20]. The CFQ consists of 25 items describing failures in attention, memory, perception, and action in everyday situations (e.g., forgetting what one intended to do or misplacing objects). Items are rated on a five-point Likert scale (“never” to “always”), yielding a total score between 0 and 100, with higher scores indicating more frequent cognitive failures. Based on general population data (mean = 32.5, SD = 11 [21,22]) a cutoff score of ≥43 (mean + 1 SD) was used to identify elevated subjective cognitive complaints, indicating a level of complaints higher than expected in the general population. A Serbian-language version of the CFQ was used. As no validated Serbian version was available, the questionnaire was translated using a forward–back translation procedure performed by bilingual researchers, followed by review by the research team to ensure conceptual equivalence with the original instrument.

Symptoms of anxiety and depression were assessed using the Hospital Anxiety and Depression Scale (HADS) [23]. The scale consists of two subscales, each with seven items rated from 0 to 3, yielding separate scores for anxiety (HADS-A) and depression (HADS-D), ranging from 0 to 21. Higher scores indicate greater symptom burden.

Health-related quality of life was evaluated with the 36-Item Short Form Health Survey (SF-36; QualityMetric, Inc., Lincoln, RI, USA) [24], a widely used generic self-report measure of perceived health status. The Serbian version of the SF-36 (version 1.0) has been validated, demonstrating excellent internal consistency (Cronbach’s α = 0.897) and adequate construct validity in the Serbian population [25]. The questionnaire comprises eight domains: Physical Functioning, Role Limitations due to Physical Problems, Bodily Pain, General Health, Vitality, Social Functioning, Role Limitations related to Emotional Problems, and Mental Health. Domain scores were transformed to a 0–100 scale, with higher scores indicating better HRQoL. Physical (PCS) and mental (MCS) component summary scores were derived from the eight domains using the standard scoring algorithm described in the SF-36 manual [26]. All questionnaires were administered face-to-face by the investigator during the one-year follow-up visit.

### 2.3. Statistical Analysis

All analyses were performed using SPSS version 26 (IBM Corp., Armonk, NY, USA). Descriptive statistics were summarized as mean ± SD or median (IQR). For comparisons of MoCA subdomains between groups, a Holm–Bonferroni correction was applied to account for multiple testing. These analyses were considered exploratory. Between-group differences (aSAH vs. controls) for MoCA, CFQ, and SF-36 domains were examined using the Mann–Whitney *U* test. Differences across demographic subgroups were assessed using the Kruskal–Wallis *H* test with Bonferroni-adjusted post hoc tests when appropriate. Categorical variables were analyzed using χ^2^ tests (with Fisher’s exact test for sparse cells). Associations among cognitive, demographic, clinical, and HRQoL variables were tested using Spearman’s *ρ*. Effect sizes were provided for nonparametric comparisons (r for Mann–Whitney; *φ*/Cramer’s *V* for χ^2^). Hierarchical multiple regression analyses were used to identify independent predictors of MoCA performance, subjective cognitive complaints and HRQoL outcomes. Given the relatively small sample size, predictors were entered in theoretically defined blocks to reduce model overfitting and to limit the number of predictors relative to the sample size. Predictors were entered hierarchically in four blocks: (1) demographic variables (age and employment), (2) clinical variables (aneurysm location and ischemia), (3) affective variables (HADS-Anxiety and HADS-Depression), and (4) cognitive variables (MoCA and CFQ). Employment status (employed, unemployed, retired) and clinical variables were treated as categorical predictors. Aneurysm location was coded as anterior versus posterior circulation, and ischemia as absent versus present. Continuous variables were entered as raw scores, and categorical predictors were dummy-coded prior to analysis. The primary HRQoL outcomes were the SF-36 physical and mental component summary scores (PCS and MCS). Analyses of individual SF-36 domains were considered exploratory and are presented in the Supplementary Material. Because SF-36 domains are conceptually and statistically interrelated and domain-level analyses were considered exploratory, formal correction for multiple comparisons was not applied; instead, results were interpreted cautiously. A two-tailed *p* < 0.05 threshold was considered statistically significant. Results with *p* values between 0.05 and 0.10 are reported as trends and interpreted cautiously. Multicollinearity was assessed using variance inflation factors (VIF) and tolerance statistics. All predictors showed acceptable values (VIF < 4.0; tolerance > 0.25), indicating no evidence of problematic multicollinearity.

## 3. Results

Sixty-four patients fulfilled the initial clinical criteria (aSAH, Hunt & Hess grades I–II, endovascular treatment), of whom 50 met the full eligibility criteria for follow-up. Two patients died during the study period, and eight eligible patients were lost to follow-up (six did not attend the scheduled visit and two declined participation due to personal circumstances), resulting in a final cohort of 40 aSAH survivors. Eighty matched controls completed the assessment. Patients who completed follow-up did not differ from those lost to follow-up in age, sex, Hunt and Hess grade, aneurysm location, or complications (all *p* > 0.05). The groups were comparable in age (*t*(78) = 0.32, *p* = 0.751) and sex (χ^2^(1) = 0.01, *p* = 0.920). Nearly all patients demonstrated good neurological recovery (mRS ≤ 1 in 92.5%), while mild residual disability (mRS = 2) was present in 7.5% of patients. Detailed demographic and clinical characteristics have been reported previously [27].

**Figure 1:**
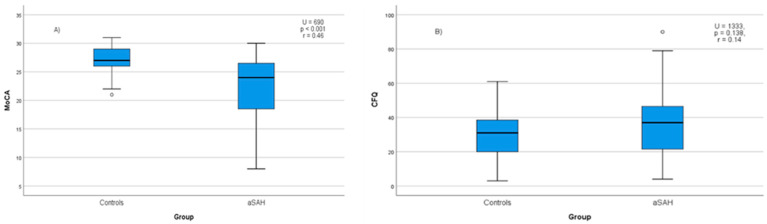
*MoCA* (**A**) and *CFQ* (**B**) scores in the aSAH and control groups.

MoCA scores were significantly lower in the aSAH group (*M* = 22.3, *SD* = 5.3) compared with controls (M = 27.2, SD = 2.2; U = 690, *p* < 0.001, *r* = 0.46; Figure 1A). Using a cutoff score of ≤22, cognitive impairment was present in 42.5% of patients and 3.8% of controls (χ^2^(1) = 26.11, *p* < 0.001, *φ* = 0.47). Patients demonstrated significantly lower scores in visuospatial/executive functions, language, delayed recall, orientation, and abstraction (all remaining significant after Holm–Bonferroni correction). In contrast, differences in the attention subdomain were smaller and did not reach statistical significance (Supplementary Table S1).

Within the patient group (Table 1), cognitive impairment was significantly more frequent in men (χ^2^(1) = 4.18, *p* = 0.040, *φ* = 0.32) and in older participants (χ^2^(2) = 6.65, *p* = 0.036, *V* = 0.41). The prevalence of impairment increased across age categories, from 16.7% in patients younger than 45 years to 77.8% among those aged ≥65 years. Education (*p* = 0.07, *φ* = 0.29) and employment status (*p* = 0.056, *φ* = 0.38) showed trend-level associations with impairment. In contrast, no demographic variable was associated with *MoCA* impairment in the control group (all *p* > 0.10).

No clinical characteristics were associated with MoCA-defined cognitive impairment. Impairment rates did not differ by Hunt & Hess grade, aneurysm location, ischemia, hydrocephalus, length of hospital stay, or premorbid comorbidity burden (all *p* > 0.05).

To further examine these associations, a hierarchical multiple regression analysis was performed with MoCA total score as the dependent variable. Demographic variables (age, sex, education, employment status, and living arrangement) explained a significant proportion of variance in MoCA performance (*R*^2^ = 0.436, adjusted *R*^2^ = 0.354, *p* = 0.001), with older age emerging as the only independent predictor of lower cognitive performance (*β* = −0.50, *p* = 0.001). The addition of clinical variables (Hunt & Hess grade, ischemia, hydrocephalus, length of hospital stay, and aneurysm location) did not significantly improve model fit (Δ*R*^2^ = 0.049, *p* = 0.731), and none of the clinical variables were independently associated with MoCA scores in the final model.

Median CFQ scores were higher and showed greater variability in aSAH survivors (Mdn = 37, IQR = 26) than in controls (Mdn = 31, IQR = 19), although this difference was not statistically significant (U = 1333, *p* = 0.138, *r* = 0.14; Figure 1B). However, when applying an established cutoff for clinically relevant complaints (CFQ ≥ 43), a significantly higher proportion of aSAH survivors met the criterion (32.5%) compared with controls (11.3%) (χ^2^(1) = 8.04, *p* = 0.005; OR = 3.80, 95% CI 1.46–9.91). Within the aSAH group, no demographic or clinical variables were significantly associated with CFQ total scores (all *p* > 0.10). In the control group, women reported significantly higher CFQ scores than men (U = 385.0, *p* = 0.001), with no other demographic associations observed.

CFQ scores were modestly and negatively correlated with MoCA performance in aSAH survivors (*ρ* = −0.33, *p* = 0.040). In a hierarchical regression analysis with CFQ total score as the dependent variable, demographic and clinical variables did not explain a significant proportion of variance. In contrast, the inclusion of MoCA scores in the final step accounted for an additional 16.6% of variance (Δ*R*^2^ = 0.166, *p* = 0.014), with lower MoCA scores independently associated with higher CFQ scores (*β* = −0.57, *p* = 0.014).

Group comparisons showed that aSAH survivors reported significantly lower HRQoL one year after ictus compared with matched controls across multiple SF-36 domains (Table 2). The largest differences were observed for physical functioning (U = 661.0, *p* < 0.001, *r* = 0.48), role-physical (U = 659.5, *p* < 0.001, *r* = 0.52), and role-emotional (U = 839.5, *p* < 0.001, *r* = 0.46). Moderate differences were also present for vitality (U = 1208.5, *p* = 0.028, *r* = 0.20) and social functioning (U = 1160.5, *p* = 0.015, *r* = 0.22). Emotional well-being and bodily pain showed no significant between-group differences (both *p* > 0.10). Overall health perceptions were significantly lower in the aSAH group (U = 1162.5, *p* = 0.014, *r* = 0.22).

Demographic and clinical variables showed only isolated associations with SF-36 domains in preliminary bivariate analyses (e.g., older age with worse Bodily Pain among aSAH survivors, and expected age- and sex-related effects in controls). Importantly, no consistent pattern across HRQoL domains emerged. Nevertheless, these variables were retained as covariates in all subsequent regression models to control for potential confounding and to isolate the unique contribution of objective and subjective cognitive measures to HRQoL.

Among aSAH survivors, higher MoCA scores were positively correlated with Mental Health (*ρ* = 0.34, *p* = 0.030), Bodily Pain (*ρ* = 0.45, *p* = 0.004), and General Health (*ρ* = 0.44, *p* = 0.005). CFQ scores correlated negatively with all SF-36 domains, with the strongest associations observed for Vitality (*ρ* = −0.79, *p* < 0.001), Social Functioning (*ρ* = −0.69, *p* < 0.001), and General Health (*ρ* = −0.69, *p* < 0.001). In controls, MoCA scores were not associated with any SF-36 domain, whereas CFQ scores correlated negatively with Physical Functioning (*ρ* = −0.28, *p* = 0.012), Role-Physical (*ρ* = −0.27, *p* = 0.015), Bodily Pain (*ρ* = −0.24, *p* = 0.030), and General Health (*ρ* = −0.29, *p* = 0.008) (Table 3).

Hierarchical regression analyses (Table 4) were conducted to identify predictors of the physical (PCS) and mental (MCS) component summaries of the SF-36. After controlling for demographic, clinical, and affective variables, the final model for PCS explained 72.2% of the variance. Employment (*β* = −0.423, *p* < 0.001) and HADS-Anxiety (*β* = −0.537, *p* = 0.002) emerged as significant independent predictors, while subjective cognitive complaints (CFQ) showed a trend toward significance (*β* = −0.287, *p* = 0.067). For MCS, the final model explained 74.6% of the variance, with HADS-Anxiety (*β* = −0.520, *p* = 0.002) and CFQ (*β* = −0.475, *p* = 0.002) identified as significant predictors, whereas employment showed a marginal association with MCS (*β* = −0.185, *p* = 0.055). Objective cognitive performance (MoCA) was not associated with either summary score.

Detailed results for individual SF-36 domains are presented in Supplementary Table S2. In exploratory domain-level analyses, subjective cognitive complaints (CFQ) remained significant predictors of Social Functioning, Role Emotional, and Vitality after controlling for HADS, whereas objective cognitive performance (MoCA) was associated with Role Emotional and Mental Health. Employment and HADS-Anxiety emerged as consistent predictors across multiple HRQoL domains.

Multicollinearity was assessed using variance inflation factors (VIFs) and tolerance statistics. All predictors showed acceptable values (VIF range 1.0–3.7; tolerance > 0.25), indicating no evidence of problematic multicollinearity. Examination of standardized residuals, leverage values, and Cook’s distance revealed no influential observations.

## 4. Discussion

This prospective cohort study examined objective cognitive performance, subjective cognitive complaints, and health-related quality of life one year after mild-grade aneurysmal subarachnoid hemorrhage. Despite favorable neurological recovery, aSAH survivors exhibited a twofold burden: persistent objective cognitive deficits, with 42.5% scoring in the impaired range on the MoCA, along with a considerable proportion reporting clinically significant subjective cognitive complaints, and substantially reduced HRQoL compared with matched controls. Crucially, a pronounced dissociation emerged between objective and subjective cognition: subjective cognitive complaints were only weakly related to objective test performance, yet showed strong and consistent associations with multiple HRQoL domains (SF-36). This pattern indicates that perceived cognitive difficulties have a greater functional impact on everyday life and perceived health than objectively measurable cognitive impairment, highlighting a critical gap between neurological recovery and patient-centered outcomes.

The nature of the objective cognitive burden was reflected in a distinct deficit profile. Beyond the high rate of global cognitive impairment (42.5% in aSAH survivors versus 3.8% in controls, MoCA ≤ 22), deficits were most pronounced in visuospatial/executive functions, language, delayed recall, orientation, and abstraction with relative sparing of attention. This profile aligns with contemporary studies identifying memory and frontal–executive functions as core vulnerabilities following aSAH [6,28]. The prevalence of impairment in our cohort is consistent with estimates commonly reported during the first postictal year, which typically range from 40% to over 70% [6,29,30]. This underscores a critical clinical insight: a favorable functional outcome, as indexed by low mRS scores, does not necessarily reflect preserved cognitive functioning, as persistent deficits may remain masked by physical independence [6]. The substantial variability in prevalence rates across the broader literature, ranging from 4% to over 70% [31,32], likely reflects methodological differences in assessment tools, timing of evaluation, and access to neurorehabilitation [33,34]. Longitudinal studies indicate that cognitive recovery is most dynamic within the first year, often plateauing after 6–12 months, with residual deficits persisting long-term in a subset of patients [14,35,36]. Accordingly, our one-year assessment point was well-suited to capture relatively stable, residual deficits rather than transient post-acute effects. Given evidence that such impairments may persist long term [37], their early and accurate identification is clinically relevant and supports the rationale for targeted cognitive rehabilitation.

The observed association between older age and lower MoCA scores reflects the expected pattern of age-related cognitive decline. Furthermore, the associations between better cognitive performance and both higher education and ongoing (vs. retired) employment support the role of cognitive reserve, whereby premorbid intellectual and occupational engagement may preserve cognitive functioning after aSAH [6,29]. Together, these findings are consistent with theoretical models of cognitive reserve, which emphasize sustained cognitive and social activity as key mechanisms supporting compensatory processes following acquired brain injury [38,39].

Contrary to clinical expectations, indicators of acute-phase severity, including Hunt & Hess grade, ischemia, hydrocephalus, aneurysm location, and length of hospital stay, were not associated with cognitive outcome at one year. This finding is in line with several previous studies [6,29,36], although results across the literature remain heterogeneous [40,41,42,43]. The lack of association suggests that long-term cognitive deficits after aSAH may be only partially explained by initial clinical severity and may instead reflect secondary or diffuse brain processes that persist despite good neurological recovery [44].

A distinct and clinically critical finding of our study was the dissociation between subjective cognitive experience and objective test performance. While mean CFQ scores did not differ significantly between groups, aSAH survivors showed higher scores with markedly greater variability, and a significantly higher proportion met the criterion for clinically relevant complaints (CFQ ≥ 43). Despite this, the overall correlation between CFQ and MoCA remained modest, underscoring the discrepancy between perceived and measured cognition. This pattern is consistent with broader evidence demonstrating weak associations between subjective complaints and neuropsychological test performance [45]. Importantly, longitudinal studies indicate that subjective cognitive complaints carry greater psychosocial relevance than objective measures, particularly for everyday functioning, return to work, and quality of life [7,28]. Accordingly, the clinical significance of subjective complaints in aSAH survivors lies not in their correspondence with test scores, but in their strong association with real-world functional outcomes.

In our sample, subjective cognitive complaints were not significantly associated with demographic or clinical characteristics, suggesting that perceived cognitive difficulties after aSAH are not readily explained by traditional indicators of disease severity or recovery course. This finding supports the notion that subjective cognitive experience represents a distinct dimension of post-aSAH outcome. The prevalence of clinically relevant subjective complaints in our cohort was lower than that reported in larger longitudinal aSAH studies, where higher rates have been observed particularly during earlier phases of recovery [7,14,15]. This discrepancy likely reflects differences in timing of assessment, sample characteristics, and methodological approaches, as well as the relatively favorable neurological outcomes of patients included in the present study.

The dissociation between objective and subjective cognition may be interpreted within a framework of network-level dysfunction following vascular brain injury. One plausible mechanism involves disrupted connectivity within the default mode network (DMN), which has been associated with both subjective cognitive complaints and objective deficits after aSAH [46,47]. Given the DMN’s role in self-referential and socio-emotional processing, its dysfunction may also contribute to impaired social reintegration and reduced quality of life [48], providing a potential neurobiological explanation for the strong association between subjective cognitive complaints and HRQoL observed in this study.

HRQoL represents a meaningful functional endpoint in survivors of aSAH. In our cohort, HRQoL was markedly reduced one year after ictus compared with healthy controls, with the largest deficits observed in domains of daily and social functioning. This finding, that favorable neurological recovery often coexists with substantially impaired quality of life, has been consistently reported [6,49,50] and underscores the limitations of relying solely on standard functional scales to capture the long-term psychosocial burden of aSAH [51]. These results highlight the need to consider HRQoL as a core, patient-centered outcome measure in post-aSAH recovery.

The homogeneity of our sample (with all patients treated endovascularly) likely minimized confounding related to treatment modality, a factor known to differentially influence QoL domains [52]. This design choice allowed a clearer isolation of the cognitive contributions to HRQoL, a focus that is particularly warranted given evidence that emotional and sleep-related symptoms are often stronger determinants of QoL than cognitive impairment per se [50,53]. Accordingly, the influence of broad clinical and demographic characteristics was attenuated in our models, contrasting with earlier studies that identified them as primary HRQoL predictors [9,12,54]. To isolate the unique contribution of cognitive factors to HRQoL, we controlled for symptoms of anxiety and depression in our regression models. After adjustment for these affective variables, subjective cognitive complaints remained independently associated with mental HRQoL, indicating that perceived cognitive difficulties contribute to long-term well-being above and beyond the influence of mood disturbance. These findings underscore that subjective cognition represents a distinct and clinically relevant dimension of post-aSAH outcome, even when affective symptoms are taken into account, and align with prior work identifying patient-reported cognitive difficulties, alongside mood and fatigue, as key long-term determinants of outcome after aSAH [12,13,55].

Correlation analyses revealed a clear pattern: perceived cognitive difficulties were strongly associated with all HRQoL domains among aSAH survivors, with the strongest relationships observed for vitality, social functioning, and general health. In contrast, objective cognitive performance showed more limited associations, reaching significance only for selected domains such as mental health, bodily pain, and general health. This pattern suggests that perceived cognitive inefficiencies may be more closely related to everyday functioning and perceived well-being than performance on brief cognitive screening measures. Importantly, these associations were attenuated but not fully eliminated after adjustment for affective symptoms in multivariable models, indicating that subjective cognitive complaints capture aspects of patient experience that extend beyond mood disturbance alone.

Exploratory domain-level analyses indicated that reduced HRQoL one year after aSAH reflects the interplay of cognitive, affective, vocational, and clinical factors. Subjective cognitive complaints and anxiety symptoms emerged as particularly relevant predictors of psychosocial aspects of HRQoL, whereas objective cognitive performance showed a more circumscribed, domain-specific influence. After adjustment for demographic, clinical, and affective variables, MoCA scores remained associated with Role Emotional and Mental Health, suggesting that cognitive performance may be associated with certain psychological dimensions of post-aSAH functioning. The unexpected negative association between MoCA and Role Emotional, despite the absence of a corresponding bivariate relationship, may reflect a statistical suppression effect related to shared variance among predictors in a relatively small sample and should therefore be interpreted cautiously. Employment status was consistently associated with several physical and role-related domains, underscoring that failure to return to work represents an important marker of broader functional limitations after aSAH.

The literature remains heterogeneous regarding the relevance of objective cognition for HRQoL outcomes. While Beallo et al. [53] reported no independent association between objective cognitive performance and HRQoL, Rass et al. [6] demonstrated that objectively defined cognitive impairment substantially increased the likelihood of reduced HRQoL one year after aSAH. Taken together, these findings suggest that objective cognitive deficits retain clinical relevance, whereas subjective cognitive difficulties may represent a more stable and functionally salient predictor of HRQoL in the chronic phase of recovery.

Collectively, these findings shift the clinical focus from what is objectively measured to what is subjectively experienced and functionally achieved. They suggest that improving quality of life after aSAH may require addressing several modifiable targets, including subjective cognitive distress, affective symptoms, vocational reintegration, and social support. From this perspective, follow-up care after aSAH may benefit from moving beyond predominantly neurological surveillance toward multidimensional rehabilitation strategies that address the broader determinants of patient-centered recovery.

Future research should further clarify the mechanisms underlying subjective cognitive complaints after aSAH, particularly the interplay between metacognitive processes, affective symptoms, and network-level brain dysfunction. Studies combining comprehensive neuropsychological assessment, neuroimaging, and longitudinal follow-up may help determine how subjective cognitive experience evolves over time and how it relates to functional recovery and participation outcomes. Such work may also inform the development of targeted rehabilitation strategies addressing both cognitive and psychosocial dimensions of post-aSAH recovery.

In conclusion, this study demonstrates a marked dissociation between objective cognitive performance and subjective cognitive complaints one year after mild-grade aneurysmal subarachnoid hemorrhage. While objective cognitive deficits were common and warrant recognition and targeted rehabilitation, subjective cognitive difficulties and anxiety symptoms emerged as key determinants of reduced health-related quality of life, particularly in the mental health domain. These findings indicate that long-term outcome after aSAH cannot be adequately captured by neurological recovery alone and emphasize that patient-reported cognitive difficulties should be routinely incorporated into outcome assessment and rehabilitation planning.

## 5. Limitations and Strengths

This study has limitations that should be considered. The relatively small sample size from a single center may limit statistical power to detect associations with less frequent clinical characteristics. Furthermore, the use of the MoCA, although a well-validated screening tool, does not allow for the detailed assessment of subtle or domain-specific deficits that would be captured by a comprehensive neuropsychological battery. In addition, both subjective cognitive complaints and HRQoL were assessed using self-report instruments administered at the same time point. While this approach is appropriate for capturing patient-perceived functioning, it may partly contribute to the observed strength of associations between these measures.

However, several key design features simultaneously constitute strengths that enhance the internal validity and clinical interpretability of our findings. First, restricting the sample to endovascularly treated patients with mild clinical grades (Hunt & Hess I–II) resulted in a clinically homogeneous cohort, minimizing confounding related to initial severity or treatment modality. Second, the sample size reflects the feasible consecutive recruitment of this specific patient population within a one-year period. Finally, the 1:2 matched case–control design improved statistical stability and effectively controlled for key demographic confounders. Thus, while acknowledging the inherent constraints of research in mild aSAH populations, these deliberate methodological choices strengthen confidence in our core conclusions regarding the dissociation between objective and subjective cognition and their differential impact on HRQoL.

## Figures and Tables

**Table 1:** Distribution of MoCA cutoff categories according to demographic characteristics.

Variable	Group	Categories	MoCA > 22 (N, %)	MoCA ≤ 22 (N, %)	χ^2^ (df)	*p*	Effect Size (*φ* or V)
Gender	aSAH	male	5 (21.7%)	9 (52.9%)	4.18 (1)	0.040	0.32
		female	18 (78.3%)	8 (47.1%)			
	Controls	male	27 (35.1%)	1 (33.3%)	0.004 (1)	0.951	0.01
		female	50 (64.9%)	2 (66.7%)			
Age	aSAH	<45	5 (83.3%)	1 (16.7%)	6.65 (2)	0.036	0.41
		45–64	16 (64.0%)	9 (36.0%)			
		≥65	2 (22.2%)	7 (77.8%)			
	Controls	<45	12 (100.0%)	0 (0.0%)	3.59 (2)	0.166	0.21
		45–64	49 (98.0%)	1 (2.0%)			
		≥65	16 (88.9%)	2 (11.1%)			
Education	aSAH	Low/inter	19 (82.6%)	17 (100.0%)	3.29 (1)	0.070	0.29
		High	4 (17.4%)	0 (0.0%)			
	Controls	Low/inter	69 (89.6%)	3 (100.0%)	0.35 (1)	0.556	0.07
		High	8 (10.4%)	0 (0.0%)			
Employment	aSAH	Employed	11 (47.8%)	4 (23.5%)	5.75 (2)	0.056	0.38
		Unemployed	8 (34.8%)	4 (23.5%)			
		Retired	4 (17.4%)	9 (52.9%)			
	Controls	Employed	57 (74.0%)	1 (33.3%)	3.52 (2)	0.172	0.21
		Unemployed	4 (5.2%)	0 (0.0%)			
		Retired	16 (20.8%)	2 (66.7%)			
Living arrangement	aSAH	With others	21 (91.3%)	16 (94.1%)	0.11 (1)	0.743	0.05
		Alone	2 (8.7%)	1 (5.9%)			
	Controls	With others	69 (89.6%)	3 (100.0%)	0.35 (1)	0.556	0.07
		Alone	8 (10.4%)	0 (0.0%)			
Comorbidities	aSAH	≥1 comorbidity	17 (54.8%)	14 (45.2%)	0.39 (1)	0.532	0.10
		None	6 (66.7%)	3 (33.3%)			
	Controls	≥1 comorbidity	38 (49.4%)	3 (100.0%)	2.97 (1)	0.085	0.19
		None	39 (50.6%)	0 (0.0%)			

Note. Values are presented as N (%). χ^2^ = chi-square test. Effect sizes are reported as *φ* for 2 × 2 tables and Cramer’s *V* for larger contingency tables. *p* values are two-tailed. MoCA = Montreal Cognitive Assessment.

**Table 2:** Group Comparison of SF-36 Domain Scores (aSAH vs. controls).

SF-36 Domain	aSAH (Mean ± SD)	Controls (Mean ± SD)	U	*p*	Effect Size (*r*)
Physical Functioning	61.88 ± 28.90	88.31 ± 13.62	661.0	<0.001	0.48
Role-Physical	41.04 ± 40.09	85.65 ± 26.52	659.5	<0.001	0.52
Role-Emotional	50.83 ± 44.65	88.40 ± 25.79	839.5	<0.001	0.46
Vitality	58.63 ± 19.01	67.91 ± 15.25	1208.5	0.028	0.20
Mental Health	67.60 ± 19.57	76.41 ± 13.48	1306.0	0.101	0.15
Social Functioning	70.31 ± 25.74	82.44 ± 17.96	1160.5	0.015	0.22
Bodily Pain	67.63 ± 32.29	75.51 ± 22.46	1453.5	0.410	0.08
General Health	61.25 ± 24.59	73.04 ± 15.47	1162.5	0.014	0.22

Note. M = mean; SD = standard deviation; U = Mann–Whitney U statistic; *r* = effect size calculated as Z/*√*N (values are presented as magnitude).

**Table 3:** Correlations between cognitive status and health-related quality of life in aSAH survivors and controls.

SF-36 Domain	aSAH MoCA *ρ* (*p*)	aSAH CFQ *ρ* (*p*)	Controls MoCA *ρ* (*p*)	Controls CFQ *ρ* (*p*)
Physical Functioning	0.30 (0.056)	−0.60 (<0.001)	0.15 (0.192)	−0.28 (0.012)
Role Physical	0.27 (0.099)	−0.40 (0.010)	0.14 (0.206)	−0.27 (0.015)
Role Emotional	0.11 (0.504)	−0.57 (<0.001)	−0.05 (0.692)	−0.10 (0.357)
Vitality	0.28 (0.082)	−0.79 (<0.001)	−0.02 (0.842)	−0.41 (<0.001)
Mental Health	0.34 (0.030)	−0.59 (<0.001)	0.12 (0.306)	−0.45 (<0.001)
Social Functioning	0.21 (0.197)	−0.69 (<0.001)	0.13 (0.239)	−0.11 (0.316)
Bodily Pain	0.45 (0.004)	−0.67 (<0.001)	0.21 (0.059)	−0.24 (0.030)
General Health	0.44 (0.005)	−0.69 (<0.001)	0.08 (0.467)	−0.29 (0.008)

Note. MoCA = Montreal Cognitive Assessment; CFQ = Cognitive Failures Questionnaire; SF-36 = 36-Item Short Form Health Survey.

**Table 4:** Hierarchical regression models predicting the physical (PCS) and mental (MCS) component summaries of the SF-36.

Domain	Predictor	*β*	*p*	Adj. R^2^
Physical Component Summary (PCS)	Employment	−0.423	<0.001	0.722
	HADS-A	−0.537	0.002	
	CFQ	−0.287	0.067	
Mental Component Summary (MCS)	HADS-A	−0.520	0.002	0.746
	CFQ	−0.475	0.002	
	Employment	−0.185	0.055	

Note. *β* = standardized regression coefficient; Adj. *R*^2^ = adjusted coefficient of determination of the final hierarchical regression model. Predictors were entered in blocks: (1) age, employment; (2) clinical variables (posterior vs. anterior circulation, ischemia); (3) affective variables (HADS-A, HADS-D); (4) cognitive variables (MoCA, CFQ). PCS = Physical Component Summary; MCS = Mental Component Summary; HADS-A = Hospital Anxiety and Depression Scale—Anxiety subscale; CFQ = Cognitive Failures Questionnaire; MoCA = Montreal Cognitive Assessment.

## Data Availability

The datasets generated and analyzed during the current study are available from the corresponding author upon request.

## Supplementary Materials

The following supporting information can be downloaded at: https://www.mdpi.com/article/10.3390/neurolint18030062/s1; Supplementary Table S1: MoCA subdomain scores in aSAH survivors and controls; Supplementary Table S2: Hierarchical multiple regression analyses predicting SF-36 domains in aSAH survivors.
