# Supplementary material for: Cognition and Health-Related Quality of Life After aSAH: The Role of Objective and Subjective Impairment
Source: Neurol Int. 2026 Mar 23;18(3):62. doi: 10.3390/neurolint18030062 (PMC13028747; doi:10.3390/neurolint18030062)
Supplement: Supplementary file 1 [file neurolint-18-00062-s001.zip › neurolint-4177352-supplementary.pdf]

Supplementary Table S1. MoCA subdomain scores in aSAH survivors and controls

MoCA subdomain	Controls Median (IQR)	Patients Median (IQR)	U	*p*	Holm *p*
Visuospatial/Executive	5 (5-5)	3.5 (2-4.5)	580	<.001	<.001
Language	5 (5-6)	4 (3.5-5)	865	<.001	<.001
Attention	6 (5-6)	6 (5-6)	1421	.259	.259
Abstraction	1 (1-2)	1 (0-2)	1172.5	.011	.022
Delayed recall	3 (3-4)	3 (0.5-3.5)	1022.5	.001	.003
Orientation	6 (6-6)	6 (5-6)	1098	<.001	<.001

Note: Data are presented as median (interquartile range, IQR). P-values were calculated using Mann–Whitney U tests and adjusted for multiple comparisons using the Holm–Bonferroni method.

Supplementary Table S2. Hierarchical multiple regression analyses predicting SF-36 domains in aSAH survivors

SF-36 Domain	Predictor	β	p	Adj. R^2
Physical Functioning	Employment	-0.300	.013	0.611
Social Functioning	HADS-A	-0.567	.005	0.626
	CFQ	-0.445	.016	
Role Physical	Employment	-0.516	<.001	0.526
Role Emotional	Employment	-0.370	.003	0.602
	Posterior circulation	-0.326	.014	
	HADS-A	-0.429	.035	
	MoCA	-0.355	.011	
	CFQ	-0.496	.010	
Vitality	Employment	-0.222	.037	0.695
	CFQ	-0.487	.004	
Mental Health	Age	0.343	.003	0.677
	Employment	-0.266	.016	
	Ischemia	0.370	.005	
	HADS-A	-0.461	.013	
	MoCA	0.272	.029	
Bodily Pain	HADS-A	-0.338	.094	0.595

SF-36 Domain	Predictor	β	p	Adj. R^2
General Health	HADS-A	-0.523	.011	0.611

Note: Variables were entered hierarchically as follows: Block 1 – demographic variables (age at SAH and employment status); Block 2 – clinical variables (posterior vs anterior circulation, ischemia); Block 3 – psychological variables (HADS-A, HADS-D); Block 4 – cognitive variables (MoCA, CFQ). β = standardized regression coefficients from the final model. Adj. R^2 = adjusted coefficient of determination for the final model. HADS-A = Hospital Anxiety and Depression Scale, Anxiety subscale; HADS-D = Hospital Anxiety and Depression Scale, Depression subscale; MoCA = Montreal Cognitive Assessment; CFQ = Cognitive Failures Questionnaire.
